# Radiomics-based outcome prediction for irinotecan-TACE in colorectal liver metastases: advanced analysis from the prospective CIREL trial

**DOI:** 10.1007/s00330-026-12573-w

**Published:** 2026-04-29

**Authors:** Zuhir Bodalal, Francisco Javier Mendoza Ferradás, Olga Maxouri, Roberto Iezzi, Aleksandar Gjoreski, Stavros Spiliopoulos, Zoltan Bansaghi, Belarmino Gonçalves, Bleranda Zeka, Nathalie Kaufmann, Julien Taieb, Regina Beets-Tan, Philippe L. Pereira, Fernando Gómez Muñoz

**Affiliations:** 1 GROW Research Institute for Oncology and Developmental Biology, Maastricht University, Maastricht, The Netherlands; 2 Department of Radiology, The Netherlands Cancer Institute, Amsterdam, The Netherlands; 3 Department of Vascular and Interventional Radiology, Hospital Universitario de Navarra, Pamplona, Spain; 4 Department of Radiological Sciences and Radiation Oncology, Institute of Advanced Interventional Radiology, Fondazione Policlinico “A. Gemelli” - IRCCS — Catholic University, Rome, Italy; 5 Department for Diagnostic and Interventional Radiology, Clinical Hospital “Acibadem-Sistina”, Skopje, North Macedonia; 6 Interventional Radiology Unit, 2nd Department of Radiology, School of Medicine, National and Kapodistrian University of Athens, Attikon University General Hospital, Athens, Greece; 7 Medical Imaging Center, Semmelweis University, Budapest, Hungary; 8 Department of Interventional Radiology, Portuguese Oncology Institute, Porto, Portugal; 9 Clinical Research Department, Cardiovascular and Interventional Radiological Society of Europe, Vienna, Austria; 10 Next Research GmbH, Vienna, Austria; 11 CARPEM comprehensive cancer center, Hepatogastroenterology and Digestive Oncology Department, Assistance Publique Hopitaux de Paris (APHP), Georges Pompidou European Hospital, Université Paris Cité, Paris, France; 12 The Netherlands Cancer Institute, Amsterdam, The Netherlands; 13 Institute of Regional Health Research, University of Southern Denmark, Odense, Denmark; 14 SLK-Kliniken Heilbronn GmbH, Center for Radiology, Minimally Invasive Therapies and Nuclear Medicine, Heilbronn, University of Heidelberg, Heidelberg, Germany; 15 Eberhard-Karls University of Tübingen, Tübingen, Germany; 16 Danube Private University, Krems, Austria; 17 Hospital Universitario y Politécnico La Fe, Valencia, Spain

**Keywords:** Radiomics, Machine learning, Colorectal liver metastases, Transarterial chemoembolization, Survival prediction

## Abstract

**Objectives:**

Transarterial chemoembolization (TACE) is a promising locoregional therapy for unresectable colorectal liver metastases, but patient selection remains challenging. We aimed to develop and validate prognostic radiomics-based machine learning models in a multicenter, prospectively collected drug-eluting microsphere TACE cohort.

**Materials and methods:**

We retrospectively analyzed 76 patients (176 lesions) from the prospective CIREL registry trial. Radiomic features were extracted from each lesion. We tested three types of imaging markers: general radiomics, intensity-based features, and lesion volume. For each, we derived baseline and delta features, reflecting the difference in feature vector values between baseline and first follow-up. Using a center-based split, we trained genetic/evolutionary machine learning models to predict survival and lesion-level response.

**Results:**

The median age of the final study population with baseline imaging was 66 years (IQR, 59–71), with 67.1% (*n* = 51) of patients identifying as male. On external validation, the baseline intensity algorithm was the only significant survival-prediction model (AUC = 0.79, 95% CI = 0.57–0.95; *p* = 0.011), outperforming baseline radiomics (AUC = 0.69, 95% CI = 0.47–0.86; *p* = 0.100) and baseline volume (AUC = 0.56, 95% CI = 0.37–0.74; *p* = 0.574). Radiomic prediction models stratified patients into distinct overall survival risk groups, with low-risk patients showing a median survival of 696 days versus 453 days (log-rank *p* = 0.0267). Integrating imaging features with laboratory variables improved lesion-level response assessment (AUC = 0.86, 95% CI = 0.66–0.99; *p* = 0.006), but did not enhance OS prediction. Lesion-level response was best identified by delta radiomics (AUC = 0.83, 95% CI = 0.63–0.97; *p* = 0.008).

**Conclusion:**

Radiomics-based machine learning models could predict overall survival in patients treated with irinotecan-TACE, offering a potential tool for patient selection.

**Key Points:**

***Question*** *Can radiomics and machine learning predict outcomes in patients with colorectal liver metastases treated with irinotecan-TACE, aiding in patient stratification and selection?*

***Findings*** *Baseline intensity features predicted overall survival (AUC = 0.79), while delta radiomics identified lesion response (AUC = 0.83) in a multicenter cohort.*

***Clinical relevance*** *These models can help identify patients likely to benefit from irinotecan-TACE and lesions most responsive to treatment. Further development would enable personalized therapy that may improve survival and reduce unnecessary interventions in non-responders.*

**Graphical Abstract:**

**Figure:**
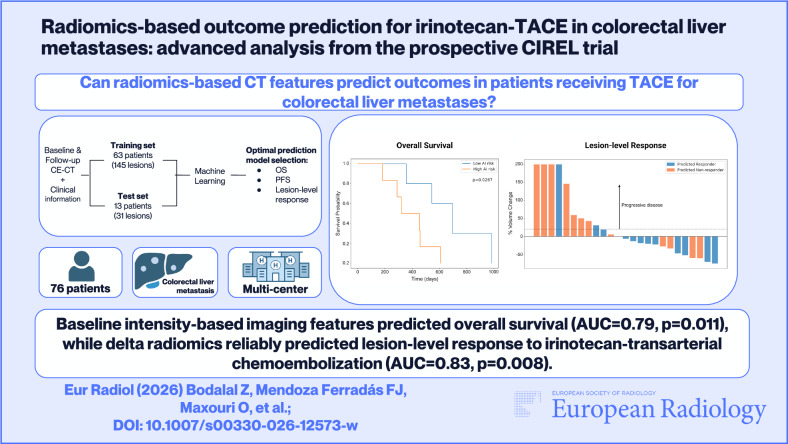

## Introduction

Colorectal liver metastases (CRLMs) are present in up to 25% of patients at diagnosis, and another 30–50% develop hepatic metastases within the first two years [1]. Surgical resection and thermal ablation can offer a potential cure for a subset of patients with resectable or “ablatable” disease, particularly when combined with systemic therapies [2, 3]. However, the majority, approximately 80%, of patients with CRLM are not eligible for these curative local treatments [4], leaving systemic therapy as the primary means of disease control. Although first- and second-line chemotherapy regimens, often paired with molecularly targeted agents, can effectively extend survival, many patients ultimately experience disease progression or endure prolonged treatment with diminishing returns and cumulative toxicity.

In patients with predominant or exclusive liver disease, transarterial chemoembolization (TACE) using irinotecan-loaded microspheres (irinotecan-TACE) has been explored as part of a broader locoregional treatment strategy [5, 6]. By enabling the sustained and targeted delivery of irinotecan to tumor-bearing hepatic segments, TACE may induce meaningful tumor responses while reducing systemic adverse effects [7]. Both retrospective and prospective data suggest that irinotecan-TACE can serve as either a salvage therapy for patients who progress on standard systemic treatments or a consolidation modality, potentially allowing maintenance therapy breaks [8, 9]. However, consensus on optimal patient selection, treatment protocols, and the optimal timing of irinotecan-TACE remains limited.

CIrse REgistry for LifePearl™ microspheres (CIREL), a pan-European, prospective observational study, was initiated to systematically gather real-world data on irinotecan-TACE in patients with CRLM [10]. Previous CIREL analyses have shed light on the safety profile of drug-eluting microsphere TACE, highlighted the incidence of adverse events, and provided new insights into procedural practices and effectiveness in Europe [4, 11, 12]. Despite these insights, significant uncertainty remains regarding the identification of suitable candidates for TACE and the characterization of which lesions are responding favorably.

Advanced medical image analysis techniques, particularly radiomics, have the potential to address these challenges. Radiomics harnesses computational algorithms to extract and quantify high-dimensional features from routinely acquired imaging data, such as contrast-enhanced CT or MRI scans. These features capture subtle spatial and intensity patterns, as well as geometric and textural morphological phenotypic properties of tumors, often exceeding the sensitivity of conventional radiological assessment [13]. Radiomics-based approaches have been associated with clinically relevant outcomes, including overall survival (OS), risk stratification, treatment response, and underlying tumor biology in oncologic settings [14–16]. In parallel, volumetric measurements, longitudinal (“delta”) imaging biomarkers, and other AI-based response-assessment algorithms have shown value in monitoring therapeutic response [17, 18], particularly when integrated with laboratory and molecular data to improve risk stratification [19–22]. Consequently, radiomics-guided image analysis holds promise for identifying patients, or individual lesions, most likely to benefit from locoregional therapies, such as TACE.

In this study, we aimed to develop and externally validate radiomics-based machine learning models for predicting OS, progression-free survival (PFS), and lesion-level response in patients with CRLM treated with irinotecan-loaded drug-eluting microsphere TACE. OS was prespecified as the primary endpoint; lesion-level response at first follow-up was analyzed as a secondary, lesion-centered endpoint, and PFS was considered exploratory. Using data from the prospective multicenter European CIREL registry, we investigated whether combining imaging-derived features and clinical variables could improve risk stratification and support personalized locoregional treatment decisions.

## Methods

### Study cohort and ethical approval

This study retrospectively analyzed prospectively collected patient data from the CIREL registry (NCT03086096), a European multicenter prospective trial assessing TACE with irinotecan-loaded LifePearl™ Microspheres (Terumo Europe NV) for colorectal cancer (CRC) liver metastases [10]. Ethical approval for the current analysis was obtained from the Institutional Review Board of the Netherlands Cancer Institute (IRBd22-317). All participants provided written informed consent at their enrolling institutions under the original CIREL protocol [11].

### Study design and patient eligibility

A total of 152 patients were enrolled across 20 tertiary care centers in 11 European countries, as previously described [4, 11, 12]. Inclusion criteria for this study included: (1) histologically confirmed CRLM, (2) at least one measurable liver metastasis, and (3) the availability of contrast-enhanced (portal venous phase) baseline CT scans suitable for volumetric and radiomic analyses. Patients were excluded if they had absent or incomplete imaging and/or outcome data, were lost to follow-up, or had poor-quality imaging. The distribution of included patients across centers is detailed in Supplementary S1. Baseline and follow-up CT scans were collected alongside clinical data (gender, age, ECOG performance status, affected lobe(s), synchronous/metachronous metastases) and laboratory parameters (liver function markers (alanine transaminase, aspartate transaminase, alkaline phosphatase, bilirubin], tumor burden markers (carcinoembryonic antigen, lactate dehydrogenase), hematological markers (neutrophils, platelets), and molecular characteristics (RAS and BRAF mutation status, mismatch repair status)). OS and PFS were recorded for all included patients. This study’s reporting followed the CheckList for EvaluAtion of Radiomics research (CLEAR) criteria [23] and the Standards for Reporting of Diagnostic Accuracy Studies (STARD) 2015 guidelines [24].

### Imaging data acquisition and segmentation

Each patient was instructed to refrain from oral intake for 2–4 hours before CT scanning; no bowel preparation was performed. Scanning was conducted on systems from Philips, Siemens Healthineers, GE Healthcare, or Canon Medical Systems, with detector configurations ranging from 16 to 320 channels (Supplementary S2). Scans were reconstructed to a slice thickness of 1–5 mm; an iodine-based contrast agent was administered intravenously before acquiring portal venous phase images approximately 70 s post-injection. The in-plane resolution was generally consistent across centers (median pixel spacing = 0.781 mm, interquartile range (IQR) = 0.706–0.872 mm). A board-certified radiologist (F.J.M.F.) with 6 years of imaging experience manually delineated liver metastases on anonymized baseline and follow-up CT scans using 3D Slicer (version 5.8.0). Lesions were identified and labeled consistently across time points to ensure spatial correspondence, using a lesion-level tracking system (e.g., mtt1, mtt2) based on anatomical location, shape, and size. Only lesions that could be confidently matched between time points were included in delta feature analyses. The radiologist was blinded to all clinical, laboratory, and outcome data.

### Radiomic feature extraction and selection

Feature extraction was performed using pyradiomics v3.1.0a1 [25], an open-source toolkit compliant with the Image Biomarker Standardization Initiative guidelines [26]. A total of 2016 radiomic features were extracted from each segmented lesion, including shape descriptors, first-order intensity statistics, and second-order texture features derived from gray level co-occurrence, run length, size zone, dependence, and neighboring tone difference matrices. Before feature extraction, all imaging data were resampled to isotropic 1 mm³ voxels using B-spline interpolation to standardize spatial resolution across diverse scanner platforms. Detailed extraction parameters, including filter configurations, discretization methods, and pre-processing, are provided in Supplementary S3. These radiomic features collectively captured the morphological, textural, and intensity-based phenotypic characteristics of each lesion. Detailed feature descriptions are further discussed in Griethuysen et al [27].

Three distinct feature sets were derived for machine learning analysis: a general radiomic signature, an intensity-based (first-order) signature, and lesion volume. For the general radiomic signature, a two-step feature reduction protocol was implemented. First, the Kruskal-Wallis test (with post-hoc Benjamini-Hochberg correction) identified and excluded features that showed a significant association with institutional origin, mitigating center-level bias. Second, pairwise Spearman correlation analysis was used to eliminate redundant features (ρ ≥ 0.90) and minimize multicollinearity. Given the cohort size and the center-based external validation design, we intentionally avoided outcome-guided pre-selection (e.g., univariate screening or penalized regression) because it would require a nested cross-validation scheme (ideally, center-stratified) to prevent information leakage and optimistic bias. Instead, we applied outcome-agnostic filtering focused on generalizability, yielding a compact radiomic feature set that prioritizes robustness across centers. The intensity signature retained only unfiltered (original) first-order features, excluding other (potentially correlated) first-order features from different filters. Delta radiomic features, which reflect longitudinal changes between baseline and first follow-up scans, were computed as differentials for all imaging markers between the two time points for each lesion.

### Machine learning modeling

**Fig. 1:**
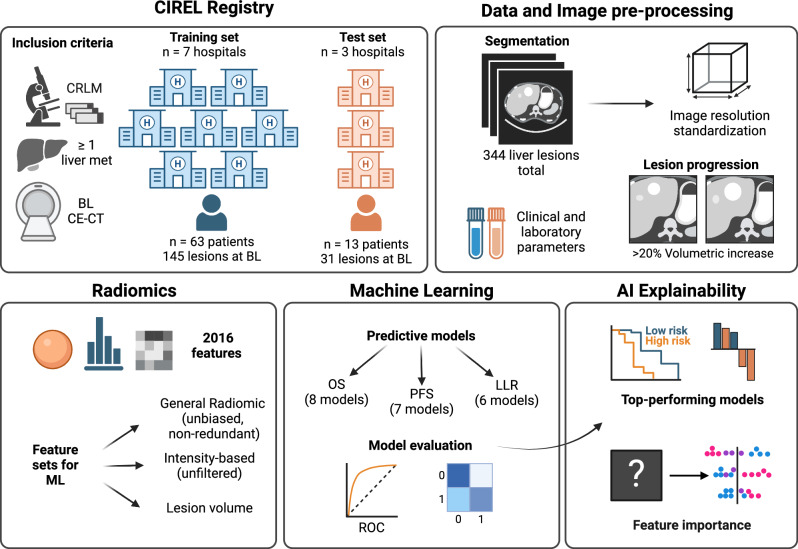
Study design overview. Graphical illustration showing the patient selection process, data acquisition, radiomic feature extraction, model development, and validation for predicting outcomes in colorectal liver metastases treated with irinotecan-TACE. BL, baseline; CE-CT, contrast-enhanced computed tomography; CRLM, colorectal liver metastases; LLR, lesion-level response; ML, machine learning; OS, overall survival; PFS, progression-free survival

Patients were divided into a training cohort (*n* = 63, 145 lesions at baseline) and an external validation cohort (*n* = 13, 31 lesions at baseline) based on the center of origin. The training cohort included patients from seven centers, while the external validation cohort consisted of patients from three centers. Three clinical endpoints were modeled: OS, PFS, and lesion-level progression. OS was prespecified as the primary endpoint; lesion-level progression (capturing lesion-level response at first follow-up) was analyzed as a secondary endpoint, and PFS was considered exploratory. Survival outcomes were dichotomized at the median survival observed in the training cohort. This was done to maintain approximate class balance and to improve model stability in our modestly sized cohort. Fixed 6-month or 12-month thresholds would have resulted in a more imbalanced distribution, which could destabilize optimization and inflate uncertainty. Lesion progression was defined as a ≥ 20% increase in volume from baseline to the first follow-up, consistent with RECIST-derived thresholds. Separate models were developed for each endpoint using baseline, delta imaging features (representing longitudinal changes), and clinical-laboratory parameters. Missing laboratory values were imputed using a univariate approach based on training-cohort distributions. For imaging-based models that demonstrated strong performance, clinical and laboratory data were integrated via early fusion (i.e., concatenation at the input level) to assess the additive value (Fig. 1).

Model optimization was performed using the open-source Tree-based Pipeline Optimization Tool (TPOT), which implements an evolutionary algorithm to automate machine learning pipeline design [28–30]. Using five-fold cross-validation on the training data, TPOT iteratively generated populations of candidate pipelines (200 per generation) over 200 generations. Each pipeline consisted of combinations of feature preprocessors, feature selectors, and classifiers, with their hyperparameters mutated across generations. The algorithm retained the top-performing pipelines from each generation and introduced stochastic variations to explore the hyperparameter space. After evaluating 40,200 unique pipeline configurations, the highest cross-validated performer was selected for external validation.

### Statistical analysis

We compared the training and test cohorts for continuous variables using two-sided Mann–Whitney *U*-tests and for categorical variables using chi-squared tests, with *p*-values < 0.05 considered statistically significant. Model performance on the external validation cohort was evaluated using bootstrapping (10,000 iterations) to derive 95% confidence intervals for the area under the receiver operating characteristic curve (AUC), precision-recall AUC (PR-AUC), accuracy, F1 score, sensitivity, specificity, Brier score, positive predictive value (PPV), and negative predictive value (NPV). We quantified intra-reader variability by re-segmenting 20 randomly selected patients and computing, for each radiomic feature, the intraclass correlation coefficient ICC(3,1) (two-way mixed-effects, consistency, single-measurement) across the two rounds. Kaplan-Meier survival analysis was conducted using an OS prediction model on the external validation cohort. Patients were stratified into ‘High’ and ‘Low’ AI risk groups based on the median of the aggregated predictions, and survival distributions were compared using the log-rank test. Feature importance was assessed with Shapley additive explanations (SHAP), which quantify the marginal contribution of each feature to model predictions [31]. All analyses were performed in Python v3.11.5 using lifelines v0.28.0, matplotlib v3.7.2, numpy v1.24.4, pandas v2.0.3, pydicom v2.4.4, pyradiomics v3.0.1a1, scipy v1.11.1, shap v0.46.0, sklearn v1.4.2, tpot v0.12.2, and trimesh v4.0.5.

## Results

### Patient characteristics

**Fig. 2:**
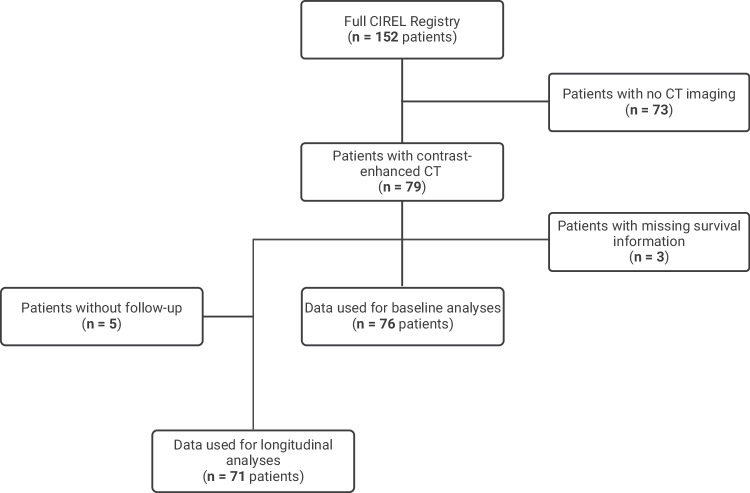
Inclusion and exclusion flowchart. A schematic depicting the selection of patients from the complete CIREL registry (*n* = 152) to the final cohorts for baseline (*n* = 76) and longitudinal analyses (*n* = 71).

Of the 152 patients initially included in the CIREL registry, 79 had suitable contrast-enhanced CT imaging for baseline volumetric and radiomic analyses. Among these, three were excluded due to incomplete survival data, resulting in 76 patients with baseline data (63 in the training set and 13 in the external validation set). This yielded a total of 145 lesions in the training cohort and 31 in the validation cohort, which were used for all predictive modeling with baseline inputs. For longitudinal (delta) analyses only, five additional patients were excluded because no follow-up scan was available, resulting in a total of 71 patients (60 in the training set and 11 in the validation set). These patients included 135 lesions in the training cohort and 25 in the validation cohort (Fig. 2). Laboratory-based analyses were restricted to patients with available imaging, survival, and laboratory data, resulting in 69 eligible patients (57 in training and 12 in validation).

The median age across the final study population with baseline imaging was 66 years (interquartile range (IQR) = 58.8–71.0), with 67.1% (*n* = 51) of patients identifying as male. The proportion of patients with an ECOG performance status of 0 was 73.7% (*n* = 56), while 71.1% (*n* = 54) had synchronous metastases at diagnosis. Right-lobe-dominant disease was observed in 47.3% (*n* = 36) of patients, and 35.5% (*n* = 27) carried a RAS mutation. The median baseline carcinoembryonic antigen level was 64.3 µg/L (IQR = 19.3–265.8), and the median lactate dehydrogenase level was 229.5 U/L (IQR = 177.5–353.3). Detailed patient characteristics are presented in Table 1 and Supplementary S4. No significant differences were observed in key demographic or clinical variables between the training and validation cohorts (all *p* > 0.05; Table 1).

**Table 1:** Baseline patient characteristics

	Total cohort	Training set	External validation	*p*
Age (median | IQR)	66.0 (58.8–71.0)	66.0 (57.5–70.5)	70.0 (62.0-74.0)	0.313
Gender (*n* | %)				
Male	51 (67.1%)	43 (68.3%)	8 (61.5%)	0.885
Female	25 (32.9%)	20 (31.7%)	5 (38.5%)	
ECOG Performance status (*n* | %)				
0	56 (73.7%)	46 (73.0%)	10 (76.9%)	0.137
1	18 (23.7%)	16 (25.4%)	2 (15.4%)	
2	1 (1.3%)	0 (0.0%)	1 (7.7%)	
3	1 (1.3%)	1 (1.6%)	0 (0.0%)	
Dominant liver lobe (*n* | %)				
Right	36 (47.4%)	30 (47.6%)	6 (46.2%)	0.072
Left	6 (7.9%)	3 (4.8%)	3 (23.1%)	
Both	34 (44.7%)	30 (47.6%)	4 (30.8%)	
Liver metastases emergence (*n* | %)				
Synchronous	54 (71.1%)	46 (73.0%)	8 (61.5%)	0.621
Metachronous	22 (28.9%)	17 (27.0%)	5 (38.5%)	
RAS mutation status (*n* | %)				
Wild type	29 (38.2%)	25 (39.7%)	4 (30.8%)	0.878
Mutated	27 (35.5%)	22 (34.9%)	5 (38.5%)	
Missing	20(26.3%)	16 (25.4%)	4 (30.8%)	
BRAF mutation status (*n* | %)				
Wild type	30 (39.5%)	27 (42.9%)	3 (23.1%)	0.211
Mutated	6 (7.9%)	6 (9.5%)	0 (0.0%)	
Missing	40 (52.6%)	29 (47.6%)	10 (76.9%)	
Affected liver segment (*n* | %) (lesion-level)				
Liver Segment: I	4 (5.3%)	3 (4.8%)	1 (7.7%)	0.386
Liver Segment: II	12 (15.8%)	10 (15.9%)	2 (15.4%)	
Liver Segment: III	11 (14.5%)	11 (17.5%)	0 (0.0%)	
Liver Segment: IV	11 (14.5%)	9 (14.3%)	2 (15.4%)	
Liver Segment: V	15 (19.7%)	13 (20.6%)	2 (15.4%)	
Liver Segment: VI	7 (9.2%)	6 (9.5%)	1 (7.7%)	
Liver Segment: VII	9 (11.8%)	5 (7.9%)	4 (30.8%)	
Liver Segment: VIII	7 (9.2%)	6 (9.5%)	1 (7.7%)	
Tumor volume, mm3 (median | IQR) (lesion-level)	13,234.4 (4140.3-96806.8)	13,800.7 (4104.0-119,461.5)	12,494.1 (7312.2–41,213.4)	0.370
Overall survival, days (median | IRQ)	362.5 (226.0–549.8)	355.0 (225.0–539.5)	453.0 (290.0–608.0)	0.486
Progression-free survival, days (median | IRQ)	143.0 (85.5–221.0)	139.0 (85.0–205.5)	219.0 (114.0–357.0)	0.140
Lesion level response (*n* | %)				
Response	91 (51.7%)	76 (52.4%)	16 (51.6%)	0.834
Non-response	85 (48.3%)	69 (47.6%)	15 (48.4%)	

Overview of demographic and clinical variables for the total cohort, training set, and external validation subset. *p*-values indicate comparisons between the training and external validation sets

### Radiomic feature extraction and selection

A total of 176 liver lesions were segmented in the baseline CT scans, and 168 matched lesions were delineated in the first follow-up. From each lesion, 2016 radiomic features were extracted, including shape descriptors, first-order intensity metrics, and various texture-derived categories. Intra-reader variability was low: across two segmentations of 20 randomly selected patients, per-feature ICC(3,1) values demonstrated high stability (median 0.975; IQR 0.899–0.991), indicating excellent repeatability. To derive the general radiomic signature, features significantly associated with the center of origin were removed, resulting in the elimination of 1915 features and the selection of 101 candidates. Among these remaining features, 62 were further excluded due to high collinearity (*ρ* ≥ 0.90), resulting in a final subset of 39 stable features used for the general radiomic signature. Only unfiltered (original) first-order radiomic features were retained for the intensity-based models, yielding 18 intensity features. The included features for each model type are presented in Supplementary S5.

### Model performance

**Fig. 3:**
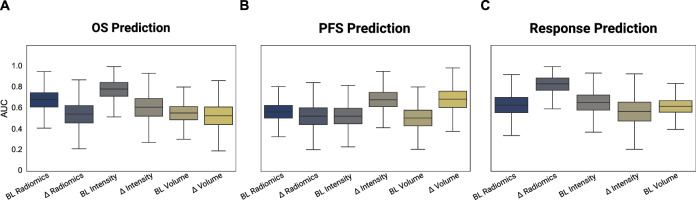
Machine learning performance for OS, PFS, and lesion-level response prediction. **A**–**C** Box plots showing the distribution of area under the ROC curve (AUC) for various baseline (BL) and delta (Δ) imaging models, including radiomics, intensity, and volume, in predicting overall survival (OS), progression-free survival (PFS), and lesion-level response

We trained and evaluated distinct, endpoint-specific machine learning models, with OS as the primary endpoint, lesion-level response as a secondary endpoint, and progression-free survival analyzed in an exploratory manner (Fig. 3A–C), using different input feature sets (baseline and delta radiomics, baseline and delta intensity signatures, baseline and delta volume, and baseline clinical/laboratory parameters). All machine learning models were built using a genetic/evolutionary algorithm on the training cohort and then evaluated on the external validation set.

### Overall survival

**Fig. 4:**
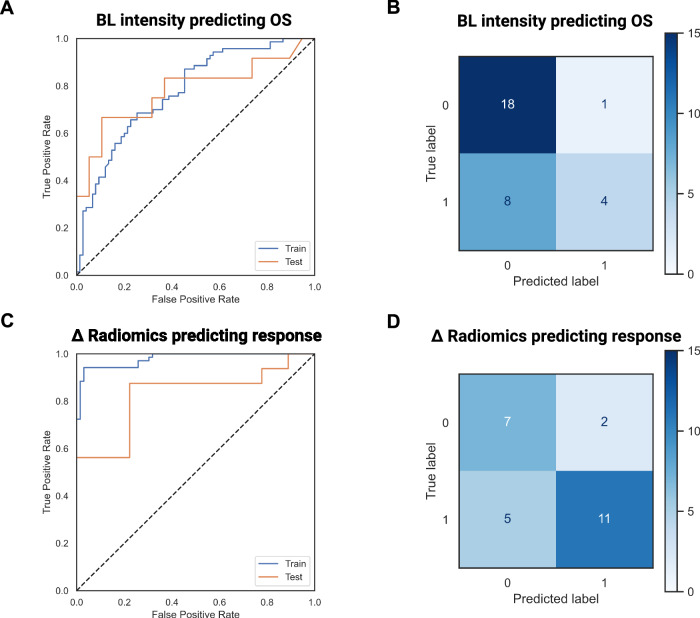
Model evaluation for top-performing models. **A**, **C** depict representative ROC curves for the top-performing BL intensity and Δ radiomics models, respectively, comparing training (blue) vs. test/external validation (orange) performance. **B**, **D** are confusion matrices illustrating the classification outcomes for overall survival (OS) and lesion-level response predictions in the external validation cohort

**Fig. 5:**
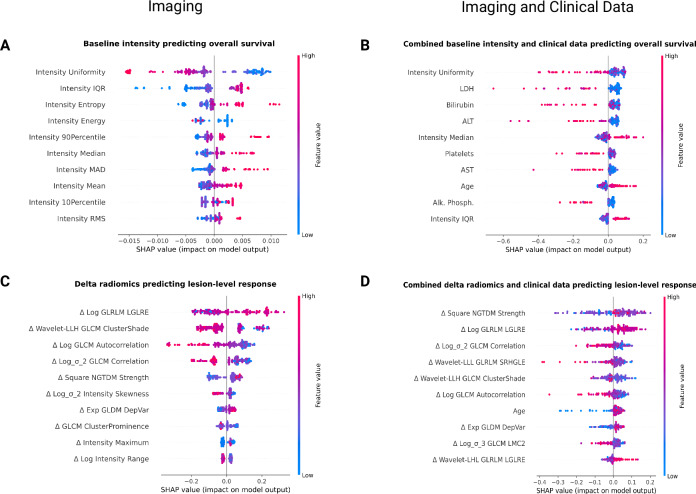
SHAP analysis of imaging-only vs combined imaging and clinical data models. **A** Key baseline intensity features that drive OS prediction in the imaging-only model. **B** Feature importance when combining baseline intensity with clinical and laboratory parameters for OS prediction. **C** Important delta radiomics features predicting lesion-level response. **D** The integrated model (delta radiomics plus clinical data) and the most influential features on lesion-level response prediction

**Fig. 6:**
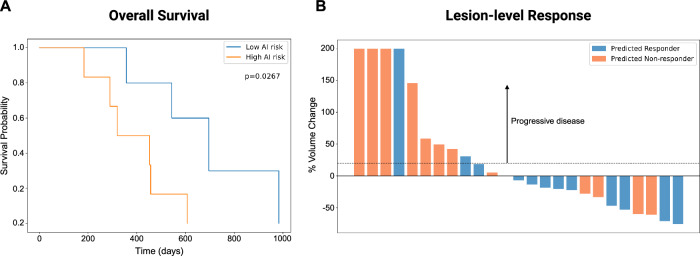
Overall survival and lesion-level response stratified by AI risk. **A** Kaplan–Meier curves comparing patients classified as high AI risk vs. low AI risk for overall survival. The log-rank test shows a significant difference (*p* = 0.0267) between the two groups. **B** Waterfall plot depicting percentage volumetric changes (*clipped at* + *200%*) in individual liver metastases, with orange bars indicating lesions predicted as non-responders and blue bars indicating predicted responders; the dashed line (20% increase) denotes the threshold for progressive disease by RECIST 1.1 criteria

Seven different modeling strategies were tested to predict OS, which was binarized at the median OS of the training set (362.5 days). On external validation, the baseline intensity algorithm emerged as the only survival prediction model achieving statistical significance (AUC = 0.79, 95% CI = 0.57–0.95; Brier score=0.24, 95% CI = 0.23-0.25; p = 0.011; Fig. 4A, B), outperforming baseline radiomics (AUC = 0.69, 95% CI = 0.47–0.86; p = 0.100) and baseline volume (AUC = 0.56, 95% CI = 0.37–0.74; *p* = 0.574). AI explainability (SHAP) analyses showed that lesions with less uniform contrast enhancement and greater signal-intensity variability within the tumor were associated with a higher likelihood of prolonged OS (Fig. 5A). In the Kaplan–Meier analysis, patients were stratified into low- and high-AI risk groups based on the median aggregated AI score of the baseline intensity model. Patients who were expected to be at lower risk according to the AI also demonstrated longer survival in the low-AI risk group (median = 696.0 days, IQR = 403.8–744.0 days) compared to the high-AI risk group (median = 453.0 days, IQR = 236.5–455.0 days, log-rank *p* = 0.0267, Fig. 6A).

Models trained on baseline intensity data also demonstrated good accuracy (0.71, 95% CI = 0.55–0.87), specificity (0.95, 95% CI = 0.82–1.00), and NPV (0.69, 95% CI = 0.50–0.86). Accounting for longitudinal changes did not enhance OS predictions: delta radiomics yielded an AUC = 0.55 (95% CI = 0.29–0.77; *p* = 0.724), delta intensity an AUC of 0.61 (95% CI = 0.36–0.83; *p* = 0.377), and delta volume an AUC = 0.53 (95% CI = 0.30–0.77; *p* = 0.806). In contrast, models trained on baseline clinical-lab data demonstrated relatively good prognostic performance (AUC = 0.86, 95% CI = 0.56–1.00) but only trended toward significance (*p* = 0.073). Table 2 summarizes key performance metrics for each OS prediction model.

**Table 2:** Overall survival predictive model performance

Input data	Endpoint	AUC	PR-AUC	F1	Accuracy	Sensitivity	Specificity	PPV	NPV	*p*	Average training AUC
Baseline radiomics	OS	0.69 (0.47–0.86)	0.81 (0.62–0.93)	0.67 (0.46–0.82)	0.61 (0.45–0.77)	0.63 (0.40–0.84)	0.58 (0.30–0.87)	0.71 (0.47–0.92)	0.50 (0.24–0.77)	0.100	0.816
Delta radiomics	OS	0.55 (0.29–0.77)	0.57 (0.32–0.83)	0.44 (0.14–0.69)	0.52 (0.32–0.72)	0.42 (0.13–0.71)	0.62 (0.33–0.87)	0.50 (0.17–0.82)	0.53 (0.27–0.79)	0.724	0.731
Baseline intensity	OS	0.79 (0.57–0.95)	0.80 (0.54–0.94)	0.46 (0.13–0.74)	0.71 (0.55–0.87)	0.33 (0.08–0.62)	0.95 (0.82–1.00)	0.83 (0.33–1.00)	0.69 (0.50–0.86)	**0.011**	0.753
Delta intensity	OS	0.61 (0.36–0.83)	0.69 (0.43–0.89)	0.56 (0.29–0.77)	0.56 (0.36–0.76)	0.54 (0.25–0.80)	0.58 (0.29–0.87)	0.58 (0.30–0.87)	0.54 (0.25–0.80)	0.377	0.765
Baseline volume	OS	0.56 (0.37–0.74)	0.65 (0.45–0.83)	0.59 (0.37–0.76)	0.55 (0.39–0.71)	0.53 (0.30–0.75)	0.58 (0.30–0.87)	0.67 (0.42–0.90)	0.44 (0.20–0.69)	0.574	0.635
Delta volume	OS	0.53 (0.30–0.77)	0.57 (0.32–0.81)	0.52 (0.22–0.75)	0.56 (0.36–0.76)	0.50 (0.21–0.80)	0.62 (0.33–0.87)	0.55 (0.23–0.85)	0.57 (0.31–0.83)	0.806	0.723
Baseline labs	OS	0.86 (0.56–1.00)	0.70 (0.32–1.00)	0.67 (0.29–0.92)	0.67 (0.42–0.92)	1.00 (1.00–1.00)	0.50 (0.14–0.86)	0.50 (0.14–0.86)	1.00 (1.00–1.00)	0.073	0.789
Merged Intensity and Lab	OS	0.62 (0.36–0.83)	0.58 (0.25–0.81)	0.48 (0.24–0.69)	0.46 (0.29–0.64)	0.80 (0.46–1.00)	0.32 (0.12–0.53)	0.35 (0.16–0.57)	0.75 (0.40–1.00)	0.349	0.916

Performance metrics for various OS models on the external validation cohort

Bold values indicate *p* < 0.05

*AUC* area under the receiver operating characteristic curve, *PR-AUC* precision-recall AUC, *PPV* F1 score, accuracy, sensitivity, specificity, positive predictive value, *NPV* negative predictive value (NPV)

### Progression-free survival

As with OS, models were assessed for their ability to predict PFS, binarized at the median PFS of the training set (143 days). No model reached statistical significance on external validation. However, delta intensity showed a near-significant discriminatory capacity (AUC = 0.68, 95% CI = 0.49–0.85; *p* = 0.077), paired with an accuracy of 0.68 (95% CI = 0.48–0.84). Models based on delta volume achieved a similarly modest performance (AUC = 0.69, 95% CI = 0.46–0.90; *p* = 0.121), while baseline radiomics (AUC = 0.57, 95% CI = 0.39–0.74; *p* = 0.472), delta radiomics (AUC = 0.53, 95% CI = 0.30–0.75; *p* = 0.844), baseline intensity (AUC = 0.53, 95% CI = 0.31–0.74; *p* = 0.828), and baseline volume (AUC = 0.51, 95% CI = 0.29–0.71; *p* = 0.953) all showed limited ability to predict PFS (Table 3).

**Table 3:** Progression-free survival predictive model performance

Input data	Endpoint	AUC	PR-AUC	F1	Accuracy	Sensitivity	Specificity	PPV	NPV	*p*	Average training AUC
Baseline radiomics	PFS	0.57 (0.39–0.74)	0.58 (0.37–0.78)	0.52 (0.25–0.73)	0.58 (0.42–0.74)	0.44 (0.19–0.69)	0.74 (0.50–0.94)	0.64 (0.33–0.91)	0.55 (0.33–0.77)	0.472	0.703
Delta radiomics	PFS	0.53 (0.30–0.75)	0.55 (0.32–0.79)	0.57 (0.32–0.76)	0.52 (0.32–0.72)	0.62 (0.33–0.87)	0.42 (0.13–0.70)	0.53 (0.29–0.79)	0.50 (0.18–0.82)	0.844	0.796
Baseline intensity	PFS	0.53 (0.31–0.74)	0.61 (0.37–0.81)	0.56 (0.33–0.74)	0.55 (0.39–0.71)	0.56 (0.31–0.81)	0.53 (0.27–0.79)	0.56 (0.31–0.80)	0.53 (0.27–0.79)	0.828	0.748
Delta intensity	PFS	0.68 (0.49–0.85)	0.61 (0.36–0.83)	0.69 (0.45–0.87)	0.68 (0.48–0.84)	0.75 (0.50–1.00)	0.62 (0.33–0.87)	0.64 (0.37–0.88)	0.73 (0.44–1.00)	0.077	0.815
Baseline volume	PFS	0.51 (0.29–0.71)	0.61 (0.38–0.81)	0.50 (0.28–0.68)	0.42 (0.26–0.58)	0.56 (0.31–0.80)	0.26 (0.06–0.50)	0.45 (0.24–0.67)	0.36 (0.09–0.67)	0.953	0.684
Delta volume	PFS	0.69 (0.46–0.90)	0.76 (0.50–0.94)	0.69 (0.46–0.86)	0.64 (0.44–0.80)	0.78 (0.50–1.00)	0.50 (0.20–0.79)	0.62 (0.37–0.86)	0.67 (0.33–1.00)	0.121	0.779
Baseline labs	PFS	0.60 (0.23–0.94)	0.55 (0.24–0.95)	0.50 (0.00–0.80)	0.50 (0.250.75)	0.60 (0.00–1.00)	0.43 (0.00–0.83)	0.43 (0.00–0.83)	0.60 (0.00–1.00)	0.639	0.832

Performance metrics for various PFS models on the external validation cohort

*AUC* area under the receiver operating characteristic curve, *PR-AUC* precision-recall AUC, *PPV* F1 score, accuracy, sensitivity, specificity, positive predictive value, *NPV* negative predictive value

### Lesion-level response

Lesion-level (non-)response was defined as a ≥ 20% increase in lesion volume from baseline to the first follow-up scan, per RECIST 1.1 criteria for progressive disease. Among the various input feature sets, delta radiomics demonstrated significant classification capacity (AUC = 0.83, 95% CI = 0.63–0.97; Brier score = 0.17, 95% CI = 0.09-0.27; *p* = 0.008), with balanced sensitivity (0.69, 95% CI = 0.44–0.91) and specificity (0.80, 95% CI = 0.50–1.00; Fig. 4C&D). SHAP analysis shows that responding lesions had lower changes in intensity skewness (Fig. 5C). Only two lesions classified by the model as responding ultimately progressed volumetrically (Fig. 6B).

Baseline intensity features performed moderately well (AUC = 0.66, 95% CI = 0.45–0.85; *p* = 0.149), while baseline radiomics (AUC = 0.63, 95% CI = 0.42–0.83; *p* = 0.228) and baseline volume (AUC = 0.62, 95% CI = 0.45–0.78; *p* = 0.172) showed only modest results with wide confidence intervals (Table 4). None of these models could significantly identify lesion-level response.

**Table 4:** Lesion-level response predictive model performance

Input data	Endpoint	AUC	PR-AUC	F1	Accuracy	Sensitivity	Specificity	PPV	NPV	*p*	Average Training AUC
Baseline radiomics	Response	0.63 (0.42–0.83)	0.67 (0.42–0.87)	0.57 (0.32–0.76)	0.61 (0.45–0.77)	0.53 (0.27–0.79)	0.69 (0.44–0.91)	0.62 (0.33–0.87)	0.62 (0.37–0.83)	0.228	0.828
Delta radiomics	Response	0.83 (0.63–0.97)	0.93 (0.79–0.99)	0.76 (0.55–0.91)	0.72 (0.52 - 0.88)	0.69 (0.44–0.91)	0.80 (0.50–1.00)	0.86 (0.62–1.00)	0.58 (0.29–0.86)	**0.008**	0.869
Baseline intensity	Response	0.66 (0.45–0.85)	0.66 (0.43–0.89)	0.73 (0.53–0.88)	0.71 (0.55–0.87)	0.75 (0.53–0.94)	0.67 (0.42–0.90)	0.71 (0.47–0.92)	0.72 (0.45–0.93)	0.149	0.675
Delta intensity	Response	0.57 (0.32–0.83)	0.71 (0.48–0.94)	0.59 (0.33–0.79)	0.56 (0.36–0.76)	0.50 (0.25–0.75)	0.67 (0.33–1.00)	0.73 (0.44–1.00)	0.43 (0.17–0.69)	0.591	0.737
Baseline volume	Response	0.62 (0.45–0.78)	0.55 (0.35–0.75)	0.67 (0.45–0.82)	0.61 (0.45–0.77)	0.80 (0.57–1.00)	0.44 (0.20–0.69)	0.57 (0.35–0.78)	0.71 (0.40–1.00)	0.172	0.701
Merged delta radiomics and labs	Response	0.86 (0.66–0.99)	0.94 (0.81–0.99)	0.79 (0.58–0.93)	0.73 (0.55–0.91)	0.80 (0.55–1.00)	0.62 (0.25–1.00)	0.80 (0.55–1.00)	0.62 (0.25–1.00)	**0.006**	0.877

Performance metrics for predicting individual lesion responses in the external validation cohort

Bold values indicate *p* < 0.05

*AUC* Area under the receiver operating characteristic curve, *PR-AUC* precision-recall AUC, *PPV* F1 score, accuracy, sensitivity, specificity, positive predictive value, *NPV* negative predictive value

### Merged analyses

Of the three modeled endpoints (OS, PFS, and lesion-level response), only baseline intensity (for OS) and delta radiomics (for lesion-level response) were statistically significant in external validation. We, therefore, examined whether combining these significant imaging models with clinical and laboratory data could enhance their performance. No combined imaging plus clinical/laboratory modeling was performed for PFS, because no imaging-based PFS model reached statistical significance on external validation.

For OS, merging baseline intensity with clinical/laboratory variables (AUC = 0.62, 95% CI = 0.36–0.83; *p* = 0.349) did not improve predictions beyond imaging alone (Table 2). The combined OS prediction model used both imaging and clinical/lab data, with lower values of contrast uniformity, lactate dehydrogenase, bilirubin, and alanine aminotransferase indicating a more prolonged OS (Fig. 5B). In contrast, for lesion-level response, integrating delta radiomics with laboratory parameters raised the AUC from 0.83 to 0.86 (95% CI = 0.66–0.99; *p* = 0.006), and most secondary metrics improved similarly, indicating that the integrated model produced more reliable classifications. The combined lesion-level response prediction model also used both delta imaging and clinical data, although only age was included among the top ten most influential features (Fig. 5D).

## Discussion

Locoregional therapy for metastatic CRC remains an evolving field, particularly for patients with liver-dominant metastatic disease who are not candidates for surgery or ablative treatments. TACE with irinotecan-loaded microspheres has emerged as a promising locoregional intervention, offering targeted drug delivery with fewer systemic side effects [32, 33]. This study aimed to improve patient selection for irinotecan-TACE using radiomics-based machine learning models derived from images and/or clinical and laboratory data based on a multicenter, prospectively collected cohort within the CIREL registry.

Our analysis identified two primary findings related to OS and lesion-level response. A machine learning model trained on baseline intensity features significantly predicted OS (AUC = 0.79, 95% CI: 0.57–0.95; *p* = 0.011) in an independent external validation cohort. These intensity-based features captured enhancement heterogeneity and lesion density patterns, which may reflect tumor vascular architecture, stromal integrity, or intralesional pressure, factors known to influence local drug delivery during TACE [34–37]. These findings align with previous studies by Song et al, who associated imaging heterogeneity with hypervascular malignant liver lesions, and Fiz et al, who reported that heterogeneous radiomic patterns in CRLMs were linked to suboptimal therapeutic outcomes [38, 39]. Tumor heterogeneity may paradoxically increase intratumoral drug distribution by disrupting vascular architecture, potentially exposing a greater number of tumor cells to the cytotoxic agent.

Secondly, at the lesion level, our analysis of delta radiomics reveals that imaging changes between baseline and the first follow-up scans can concurrently classify volumetric progression and response. Unlike a temporal prediction based solely on baseline characteristics, our approach capitalized on dynamic changes in radiomic features, such as shifts in intensity skewness, to determine whether a lesion progressed at the first follow-up. This distinction addresses a key concern: rather than predicting a future outcome from a static baseline, the delta radiomics model functions as a concurrent classifier. It quantitatively characterizes the lesion’s evolving biology following treatment. By identifying biologically non-responsive lesions despite minimal volumetric changes, the model could guide clinicians in deciding whether to continue TACE or explore alternative therapies. For instance, a lesion with stable volume but significant radiomic changes might indicate early biological resistance, prompting earlier reassessment or alternative treatments. It may also support research into the mechanistic effects of TACE by linking radiomic changes to biological processes such as angiogenesis and tumor viability, which could inform the development of biomarkers. Future studies could explore whether delta radiomics at early follow-up predicts progression at later time points, potentially offering a predictive tool for longer-term outcomes.

In our cohort, lesions with minimal changes in intensity skewness were more likely to remain controlled, suggesting that stable intensity distributions may indicate limited vascular or compositional shifts following TACE. We hypothesize that significant fluctuations in intensity skewness reflect aggressive biological behavior or rapid microscopic proliferation, as evidenced by changes in contrast uptake. Additionally, lesions with stable intensity profiles may exhibit better-preserved microarchitecture and less active neovascularization or necrosis, which could minimize volumetric expansion under TACE-induced stress.

While the associations between imaging-derived models and both OS and lesion-level response were promising, integrating clinical and laboratory parameters produced divergent effects. Incorporating clinical and laboratory data into the baseline intensity model did not enhance OS predictions. In contrast, adding these variables to the delta radiomics model improved lesion-level response assessment. The operating-point metrics illustrate different trade-offs. For OS, the merged intensity plus laboratory model favored sensitivity at the expense of specificity, whereas the imaging-only baseline intensity OS model favored specificity at the expense of sensitivity. Clinically, a high-sensitivity operating point may be conceptually useful for triage-oriented risk stratification, but it does not imply clinical readiness and would require prospective evaluation and threshold optimization.

The lack of improvement after adding laboratory variables may reflect limitations of simple early fusion (feature concatenation). Early fusion is a convenient strategy, but it can underperform when modalities are heterogeneous, when one modality is comparatively noisy or weakly informative, or when the combined feature space makes optimization and regularization more challenging [40, 41]. In our study, these factors likely contributed to the observed reduction in OS discrimination after laboratory variables were added. In clinical settings, laboratory panels often contain missing values and measurement variability. Reliance on imputation and naive concatenation can introduce additional noise and bias, potentially reducing generalization. These considerations are well recognized in multimodal learning, where alternative fusion strategies (e.g., intermediate or late fusion, and approaches explicitly designed for incomplete modalities) are being examined to enhance robustness [42]. Overall, these findings highlight that multimodal integration must be tailored to the endpoint and data context rather than assumed to be universally beneficial.

Another possible explanation is that OS in metastatic CRC is influenced by multiple systemic factors, including post-progression treatments and extrahepatic disease status, which may not be fully captured by hepatic imaging and laboratory data. These findings suggest that lesion-level response may be more tightly linked to imaging-based local features. In contrast, OS is influenced by broader systemic factors, including subsequent lines of therapy. For the more focused, lesion-specific question of whether liver metastases respond to TACE, patient-level variables such as age and inflammatory markers may further refine interpretation of radiologic changes within the broader clinical context.

Although none of the PFS models reached statistical significance on external validation, the strongest trends were observed for delta intensity (AUC 0.68; *p* = 0.077) and delta volume (AUC 0.69; *p* = 0.121), suggesting that early post-treatment changes may still carry information relevant to subsequent progression. We note that PFS is particularly sensitive to the timing and frequency of imaging assessments because progression is interval-censored between scans; variation in surveillance intervals can bias the observed PFS and increase noise, especially in small cohorts [43, 44]. In addition, progression calls depend on measurement and reader variability within RECIST-based response assessment [45, 46], which can further attenuate signal in multicenter registry data. Importantly, the lack of robust PFS prediction constrains interpretation of these models as directly predictive of treatment efficacy and supports their primary interpretation as prognostic or risk-stratification tools rather than treatment-effect predictors.

Emerging strategies in imaging-based outcome prediction may further complement the approaches explored in this study. Longitudinal body composition analysis has shown promise for capturing temporal dynamics of disease progression during locoregional and systemic therapy. For instance, longitudinal imaging has been shown to identify cachexia trajectories associated with inferior survival in patients with hepatocellular carcinoma receiving combined immunotherapy and targeted agents [47]. Similarly, multi-timepoint imaging analysis using AI-driven frameworks can extract prognostic information from serial scans that single-timepoint models may miss [48]. In parallel, habitat-based radiomics, which partitions tumors into spatially distinct subregions via voxel-wise clustering, has been shown to decode intratumoral heterogeneity and predict treatment response in patients undergoing TACE combined with systemic therapy [49]. Although these studies were conducted in hepatocellular carcinoma rather than CRLMs, the underlying principles of capturing temporal change and spatial heterogeneity are broadly applicable and represent promising directions for future radiomics research in the TACE setting.

Despite these insights, our study has a number of limitations. The external validation cohort was relatively modest, constrained by the challenges of assembling multi-institutional real-world data from patients undergoing TACE. Specifically, the external validation cohort included 13 patients (31 baseline lesions), which increases uncertainty around performance estimates and limits generalizability. This limitation primarily affects the precision of the reported metrics, as reflected by wider confidence intervals, rather than the internal validity of the modeling framework or the independence of the external validation strategy. As such, the present findings should be interpreted as preliminary and requiring confirmation in larger cohorts focused on model implementation. Nevertheless, using prospectively collected data, we demonstrate a prognostic/predictive signal that can be harnessed and serves as the foundation for future larger studies aimed at training more robust models specialized in TACE outcome prediction. Additionally, we acknowledge that patient selection and scheduling practices vary significantly across Europe. We sought to mitigate the risk of overfitting by using stringent machine learning practices, including cross-validation during training, bootstrapping to estimate confidence intervals, and splitting the cohort by center to ensure a fully independent external validation set. However, more extensive studies with larger multicenter cohorts are needed to develop more robust algorithms. Another potential constraint is the heterogeneity of imaging acquisition and reconstruction parameters. We addressed this issue by resampling voxels and excluding features strongly associated with center effects; however, some residual heterogeneity may persist. Manual segmentation, although standard in radiomics, is time-consuming and subject to inter-observer variability. To maintain consistency, we relied on a single experienced radiologist, who was blinded to the outcomes, and intra-reader repeatability was high. Automated or semi-automated segmentation methods could enhance scalability and reduce annotation burden in future broader-scale research. Still, they will require rigorous validation against multi-reader reference segmentations and assessment of how segmentation variability propagates to radiomic features and downstream model performance. Moreover, our approach involved a retrospective analysis of a prospective registry, limiting the granularity of specific clinical data points and tumor molecular profiles. Missing laboratory data necessitated imputation, though complete data collection would be ideal. Finally, we binarized OS and PFS at the training-set medians to support class balance and stable modeling; this simplifies the analysis and necessarily discards time-to-event information, representing a trade-off between clinical granularity and modeling stability in a limited cohort. However, the training-set median OS was 362.5 days, which is close to one year, so a 12-month OS cut-off would have been very similar. Future larger studies should evaluate time-to-event approaches and clinically anchored time horizons.

Such image-based AI models are intended to support risk stratification and hypothesis generation. For example, they could aid multidisciplinary team discussions by identifying patients at higher risk of poor survival and by complementing early lesion-level response assessment at first follow-up. Clinical judgment is always essential when interpreting model outputs or demonstrating treatment benefit. Assessing the clinical utility of future AI-based systems will require prospective evaluation of model-guided decision-making and downstream patient outcomes in an impact study (preferably comparative and, where appropriate, cluster-randomized), alongside transparent reporting and explicit risk-of-bias assessment [50, 51].

In conclusion, this study highlights the promise of radiomics-based machine learning models in enhancing risk stratification and treatment selection for patients with CRLM undergoing irinotecan-TACE. Baseline intensity features, together with early longitudinal changes in lesion morphology, offer a non-invasive framework for predicting patient outcomes. With validation in larger, standardized imaging datasets, these radiomics models may support more personalized locoregional treatment strategies, ultimately leading to improved clinical outcomes.

## Supplementary information

ELECTRONIC SUPPLEMENTARY MATERIAL

## Abbreviations

AUC: Area under the receiver operating characteristic curve
CLEAR: CheckList for EvaluAtion of Radiomics research
CIREL: CIrse REgistry for LifePearl™ microspheres
CRC: Colorectal cancer
CRLMs: Colorectal liver metastases
IQR: Interquartile range
NPV: Negative predictive value
OS: Overall survival
PPV: Positive predictive value
PR-AUC: Precision-recall AUC
PFS: Progression-free survival
SHAP: Shapley additive explanations
STARD: Standards for Reporting of Diagnostic Accuracy Studies
TACE: Transarterial chemoembolization
TPOT: Tree-based pipeline optimization tool

## References

[1] Patel RK, Rahman S, Schwantes IR et al (2023) Updated management of colorectal cancer liver metastases: scientific advances driving modern therapeutic innovations. Cell Mol Gastroenterol Hepatol 16:881–894. 10.1016/j.jcmgh.2023.08.012 37678799 10.1016/j.jcmgh.2023.08.012 PMC10598050

[2] Ou S, Xu R, Li K et al (2018) Radiofrequency ablation with systemic chemotherapy in the treatment of colorectal cancer liver metastasis: a 10-year single-center study. Cancer Manag Res 10:5227–5237. 10.2147/CMAR.S170160 30464620 10.2147/CMAR.S170160 PMC6217171

[3] Beg MS, Adhami F, Xuan L et al (2014) Liver-directed therapies for colorectal cancer liver metastasis (CLRM): a surveillance, epidemiology, and end results (SEER)-medicare analysis. J Clin Oncol 32:577–577. 10.1200/jco.2014.32.3_suppl.577

[4] Helmberger T, Lucatelli P, Pereira PL et al (2022) Safety, feasibility and technical considerations from a prospective, observational study-CIREL: Irinotecan-TACE for CRLM in 152 patients. J Clin Med 11:6178. 10.3390/jcm11206178 36294499 10.3390/jcm11206178 PMC9604674

[5] Van Cutsem E, Cervantes A, Adam R et al (2016) ESMO consensus guidelines for the management of patients with metastatic colorectal cancer. Ann Oncol 27:1386–1422. 10.1093/annonc/mdw235 27380959 10.1093/annonc/mdw235

[6] Gruber-Rouh T, Naguib NNN, Eichler K et al (2014) Transarterial chemoembolization of unresectable systemic chemotherapy-refractory liver metastases from colorectal cancer: long-term results over a 10-year period: TACE of Liver Metastases from Colorectal Cancer. Int J Cancer 134:1225–1231. 10.1002/ijc.28443 23960002 10.1002/ijc.28443

[7] Ishikawa T, Imai M, Sato R et al (2023) Prognostic value of TACE with irinotecan-loaded drug-eluting beads (DEBIRI) in patients with liver metastases from unresectable colorectal cancer. Anticancer Res 43:3647–3651. 10.21873/anticanres.16545 37500124 10.21873/anticanres.16545

[8] Fiorentini G, Sarti D, Nardella M et al (2020) Chemoembolization alone or associated with bevacizumab for therapy of colorectal cancer metastases: Preliminary results of a randomized study. In Vivo 34:683–686. 10.21873/invivo.11824 32111770 10.21873/invivo.11824 PMC7157852

[9] Pernot S, Pellerin O, Artru P et al (2020) Intra-arterial hepatic beads loaded with irinotecan (DEBIRI) with mFOLFOX6 in unresectable liver metastases from colorectal cancer: a Phase 2 study. Br J Cancer 123:518–524. 10.1038/s41416-020-0917-4 32507854 10.1038/s41416-020-0917-4 PMC7435188

[10] Pereira PL, Arnold D, de Baère T et al (2020) A multicentre, international, observational study on transarterial chemoembolisation in colorectal cancer liver metastases: Design and rationale of CIREL. Dig Liver Dis 52:857–861. 10.1016/j.dld.2020.05.051 32620520 10.1016/j.dld.2020.05.051

[11] Pereira PL, Iezzi R, Manfredi R et al (2021) The CIREL cohort: A prospective controlled registry studying the real-life use of irinotecan-loaded chemoembolisation in colorectal cancer liver metastases: Interim analysis. Cardiovasc Radiol 44:50–62. 10.1007/s00270-020-02646-8 10.1007/s00270-020-02646-8 PMC7728640 32974773

[12] Arnold D, Pereira PL, Iezzi R et al (2025) Transarterial chemoembolisation with irinotecan (irinotecan-TACE) as salvage or post-inductive therapy for colorectal cancer liver metastases: effectiveness results from the CIREL study. ESMO Open 10:104292. 10.1016/j.esmoop.2025.104292 39954388 10.1016/j.esmoop.2025.104292 PMC11872576

[13] Bodalal Z, Wamelink I, Trebeschi S, Beets-Tan RGH (2021) Radiomics in immuno-oncology. Immunooncol Technol 9:100028. 10.1016/j.iotech.2021.100028 35756864 10.1016/j.iotech.2021.100028 PMC9216715

[14] Bera K, Braman N, Gupta A et al (2022) Predicting cancer outcomes with radiomics and artificial intelligence in radiology. Nat Rev Clin Oncol 19:132–146. 10.1038/s41571-021-00560-7 34663898 10.1038/s41571-021-00560-7 PMC9034765

[15] Hong EK, Bodalal Z, Landolfi F et al (2022) Identifying high-risk colon cancer on CT an a radiomics signature improve radiologist’s performance for T staging? Abdom Radiol (NY) 47:2739–2746. 10.1007/s00261-022-03534-0 35661244 10.1007/s00261-022-03534-0

[16] Bodalal Z, Hong EK, Trebeschi S et al (2024) Non-invasive CT radiomic biomarkers predict microsatellite stability status in colorectal cancer: a multicenter validation study. Eur Radiol Exp 8:98. 10.1186/s41747-024-00484-8 39186200 10.1186/s41747-024-00484-8 PMC11347521

[17] Peisen F, Gerken A, Hering A et al (2024) Can delta radiomics improve the prediction of best overall response, progression-free survival, and overall survival of melanoma patients treated with immune checkpoint inhibitors. Cancers (Basel) 16:2669. 10.3390/cancers16152669 39123397 10.3390/cancers16152669 PMC11312160

[18] Abbas E, Fanni SC, Bandini C et al (2023) Delta-radiomics in cancer immunotherapy response prediction: A systematic review. Eur J Radiol Open 11:100511. 10.1016/j.ejro.2023.100511 37520768 10.1016/j.ejro.2023.100511 PMC10371799

[19] Yeghaian M, Bodalal Z, Tareco Bucho TM et al (2024) Integrated noninvasive diagnostics for prediction of survival in immunotherapy. Immunooncol Technol 24:100723. 10.1016/j.iotech.2024.100723 39185322 10.1016/j.iotech.2024.100723 PMC11342748

[20] Vanguri RS, Luo J, Aukerman AT et al (2022) Multimodal integration of radiology, pathology and genomics for prediction of response to PD-(L)1 blockade in patients with non-small cell lung cancer. Nat Cancer 3:1151–1164. 10.1038/s43018-022-00416-8 36038778 10.1038/s43018-022-00416-8 PMC9586871

[21] Yang M, Ma J, Zhang C et al (2024) Multimodal data deep learning method for predicting symptomatic pneumonitis caused by lung cancer radiotherapy combined with immunotherapy. Front Immunol 15:1492399. 10.3389/fimmu.2024.1492399 39845959 10.3389/fimmu.2024.1492399 PMC11751032

[22] Yeghaian M, Bodalal Z, van den Broek D et al (2024) Multimodal integration of longitudinal noninvasive diagnostics for survival prediction in immunotherapy using deep learning. J Am Med Inform Assoc. 32:1267–1275 10.1093/jamia/ocaf074 PMC12277703 40418276

[23] Kocak B, Baessler B, Bakas S et al (2023) CheckList for EvaluAtion of Radiomics research (CLEAR): a step-by-step reporting guideline for authors and reviewers endorsed by ESR and EuSoMII. Insights Imaging 14:75. 10.1186/s13244-023-01415-8 37142815 10.1186/s13244-023-01415-8 PMC10160267

[24] Cohen JF, Korevaar DA, Altman DG et al (2016) STARD 2015 guidelines for reporting diagnostic accuracy studies: explanation and elaboration. BMJ Open 6:e012799. 10.1136/bmjopen-2016-012799 28137831 10.1136/bmjopen-2016-012799 PMC5128957

[25] PyRadiomics (2025) Welcome to PyRadiomics documentation!—pyradiomics v3.1.0rc2.post5+g6a761c4 documentation. https://pyradiomics.readthedocs.io/en/latest/. Accessed 24 Jul 2025

[26] Zwanenburg A, Vallières M, Abdalah MA et al (2020) The image biomarker standardization initiative: Standardized quantitative radiomics for high-throughput image-based phenotyping. Radiology 295:328–338. 10.1148/radiol.2020191145 32154773 10.1148/radiol.2020191145 PMC7193906

[27] van Griethuysen JJM, Fedorov A, Parmar C et al (2017) Computational radiomics system to decode the radiographic phenotype. Cancer Res 77:e104–e107. 10.1158/0008-5472.CAN-17-0339 29092951 10.1158/0008-5472.CAN-17-0339 PMC5672828

[28] Olson RS, Urbanowicz RJ, Andrews PC et al (2016) Automating biomedical data science through tree-based pipeline optimization. In: Squillero, G., Burelli, P. (eds) Applications of Evolutionary Computation. EvoApplications 2016. Lecture Notes in Computer Science(), vol 9597. Springer, Cham. 10.1007/978-3-319-31204-0_9

[29] Olson RS, Bartley N, Urbanowicz RJ, Moore JH (2016) Evaluation of a tree-based pipeline optimization tool for automating data science. Association for Computing Machinery, New York, NY, USA. pp 485–492. 10.1145/2908812.2908918

[30] Le TT, Fu W, Moore JH (2020) Scaling tree-based automated machine learning to biomedical big data with a feature set selector. Bioinformatics 36:250–256. 10.1093/bioinformatics/btz470 31165141 10.1093/bioinformatics/btz470 PMC6956793

[31] Lundberg S, Lee S-I (2017) A unified approach to interpreting model predictions. Preprint at https://arxiv.org/abs/1705.07874

[32] Malagari K, Kiakidis T, Moschouris H et al (2023) Prospective series of transarterial chemoembolization of metastatic colorectal cancer to the liver with 30-60 μm microspheres loaded with irinotecan. Cardiovasc Radiol 46:880–890. 10.1007/s00270-023-03446-6 10.1007/s00270-023-03446-6 37337059

[33] Zhao G, Liu S, Zhang Y et al (2022) Irinotecan eluting beads-transarterial chemoembolization using Callispheres® microspheres is an effective and safe approach in treating unresectable colorectal cancer liver metastases. Ir J Med Sci 191:1139–1145. 10.1007/s11845-021-02629-9 34264426 10.1007/s11845-021-02629-9 PMC9135896

[34] Hasdemir DB, Dávila LA, Schweitzer N et al (2017) Evaluation of CT vascularization patterns for survival prognosis in patients with hepatocellular carcinoma treated by conventional TACE. Diagn Interv Radiol 23:217–222. 10.5152/dir.2016.16006 28256449 10.5152/dir.2016.16006 PMC5411003

[35] Liu K, Zhang X, Xu W et al (2017) Targeting the vasculature in hepatocellular carcinoma treatment: Starving versus normalizing blood supply. Clin Transl Gastroenterol 8:e98. 10.1038/ctg.2017.28 28617447 10.1038/ctg.2017.28 PMC5518951

[36] Jaroch DB, Liu Y, Kim AY et al (2025) Pressure-enabled drug delivery significantly increases intra-arterial delivery of embolic microspheres to liver tumors in a porcine model. J Vasc Interv Radiol 36:499–504.e1. 10.1016/j.jvir.2024.11.014 39586533 10.1016/j.jvir.2024.11.014

[37] Zhang YJ, Chen MS, Chen Y et al (2021) Long-term outcomes of transcatheter arterial chemoembolization combined with radiofrequency ablation as an initial treatment for early-stage hepatocellular carcinoma. JAMA Netw Open 4:e2126992. 10.1001/jamanetworkopen.2021.26992 34570206 10.1001/jamanetworkopen.2021.26992 PMC8477266

[38] Fiz F, Viganò L, Gennaro N et al (2020) Radiomics of liver metastases: a systematic review. Cancers (Basel) 12:2881. 10.3390/cancers12102881 33036490 10.3390/cancers12102881 PMC7600822

[39] Song S, Li Z, Niu L et al (2019) Hypervascular hepatic focal lesions on dynamic contrast-enhanced CT: preliminary data from arterial phase scans texture analysis for classification. Clin Radiol 74:653.e11–653.e18. 10.1016/j.crad.2019.05.010 31208725 10.1016/j.crad.2019.05.010

[40] Baltrušaitis T, Ahuja C, Morency L-P (2019) Multimodal machine learning: a survey and taxonomy. IEEE Transactions on Pattern Analysis and Machine Intelligence. vol. 41, pp 423-443. 10.1109/TPAMI.2018.2798607 10.1109/TPAMI.2018.2798607 29994351

[41] Ramachandram D, Taylor GW (2017) Deep multimodal learning: a survey on recent advances and trends. IEEE Signal Process Mag 34:96–108. 10.1109/msp.2017.2738401

[42] Bayoudh K, Knani R, Hamdaoui F, Mtibaa A (2022) A survey on deep multimodal learning for computer vision: advances, trends, applications, and datasets. Vis Comput 38:2939–2970. 10.1007/s00371-021-02166-7 34131356 10.1007/s00371-021-02166-7 PMC8192112

[43] Panageas KS, Ben-Porat L, Dickler MN et al (2007) When you look matters: the effect of assessment schedule on progression-free survival. J Natl Cancer Inst 99:428–432. 10.1093/jnci/djk091 17374832 10.1093/jnci/djk091

[44] Zeng L, Cook RJ, Wen L, Boruvka A (2015) Bias in progression-free survival analysis due to intermittent assessment of progression. Stat Med 34:3181–3193. 10.1002/sim.6529 26011411 10.1002/sim.6529 PMC4744753

[45] Tareco Bucho TM, Petrychenko L, Abdelatty MA et al (2024) Reproducing RECIST lesion selection via machine learning: Insights into intra and inter-radiologist variation. Eur J Radiol Open 12:100562. 10.1016/j.ejro.2024.100562 38660370 10.1016/j.ejro.2024.100562 PMC11039940

[46] Tareco Bucho TM, Tissier RLM, Groot Lipman KBW et al (2024) How does target lesion selection affect RECIST? A computer simulation study. Invest Radiol 59:465–471. 10.1097/RLI.0000000000001045 37921780 10.1097/RLI.0000000000001045

[47] Jin Z-C, Zhou J-W, Chen J-J et al (2024) Longitudinal body composition identifies hepatocellular carcinoma with cachexia following combined immunotherapy and target therapy (CHANCE2213. J Cachexia Sarcopenia Muscle 15:2705–2716. 10.1002/jcsm.13615 39604073 10.1002/jcsm.13615 PMC11634469

[48] Zou C, Mankowski W, Pantalone L et al (2026) Transformer-based fusion of longitudinal multimodal radiomic features from chest radiography and CT in COVID-19. Radiol Artif Intell e240218. 10.1148/ryai.240218 10.1148/ryai.240218 41848416

[49] Jin Z-C, Wei J, Xiao Y-D et al (2025) Decoding tumor heterogeneity with imaging biomarkers predicts response to TACE plus immunotherapy and targeted therapy in HCC (CHANCE2204). Hepatology. 10.1097/HEP.0000000000001593 10.1097/HEP.0000000000001593 PMC13668028 41213031

[50] Kappen TH, van Klei WA, van Wolfswinkel L et al (2018) Evaluating the impact of prediction models: lessons learned, challenges, and recommendations. Diagn Progn Res 2:11. 10.1186/s41512-018-0033-6 31093561 10.1186/s41512-018-0033-6 PMC6460651

[51] Collins GS, Moons KGM, Dhiman P et al (2024) TRIPOD+AI statement: updated guidance for reporting clinical prediction models that use regression or machine learning methods. BMJ 385:e078378. 10.1136/bmj-2023-078378 38626948 10.1136/bmj-2023-078378 PMC11019967

