# Supplementary material for: Radiomics-based outcome prediction for irinotecan-TACE in colorectal liver metastases: advanced analysis from the prospective CIREL trial
Source: Eur Radiol. 2026 Apr 29;36(9):6981–96. doi: 10.1007/s00330-026-12573-w (PMC13451268; doi:10.1007/s00330-026-12573-w)
Supplement: Supplementary file 1 [file 330_2026_12573_MOESM1_ESM.pdf]

Radiomics-Based Outcome Prediction for Irinotecan-TACE in Colorectal Liver Metastases: Advanced Analysis from the Prospective CIREL Trial

ELECTRONIC SUPPLEMENTARY MATERIAL

Supplementary S1. Distribution of participating centers. Overview of the number of patients and lesions in the final included cohort (centers anonymized for GDPR), specifying which were assigned to the training and external validation sets.

Country	Center Name	Train/Test	Patients (n|%)	Lesions (n|%)
Portugal	CENTER 1	Train	6 (7.9%)	14 (8.0%)
Greece	CENTER 2	Train	5 (6.6%)	9 (5.1%)
North Macedonia	CENTER 3	Train	27 (35.5%)	62 (35.2%)
Hungary	CENTER 4	Train	21 (27.6%)	50 (28.4%)
Italy	CENTER 5	Train	2 (2.6%)	6 (3.4%)
Spain	CENTER 6	Train	1 (1.3%)	1 (0.6%)
Italy	CENTER 7	Train	1 (1.3%)	3 (1.7%)
Spain	CENTER 8	Test	2 (2.6%)	6 (3.4%)
Italy	CENTER 9	Test	10 (13.2%)	22 (12.5%)
Russia	CENTER 10	Test	1 (1.3%)	3 (1.7%)

Supplementary S2. CT image acquisition parameters. Summary of in-plane resolution, slice thickness, kVp, and scanner systems in the included cohort.

**In-plane resolution (median | IQR)**	0.781 (0.706–0.872)
**Slice thickness (median | IQR)**	3.0 (1.25–3.0)
**kVp (median | IQR)**	120 (120–120)
**CT scanners (n | %)**	
Ingenuity CT	16 (21.05%)
BrightSpeed	16 (21.05%)
SOMATOM Definition AS+	7 (9.21%)
LightSpeed VCT	5 (6.58%)
SOMATOM Definition Flash	5 (6.58%)
Brilliance 16P	5 (6.58%)
Aquilion	3 (3.95%)
SOMATOM go.Up	3 (3.95%)
Revolution EVO	3 (3.95%)
Brilliance 16	3 (3.95%)
Discovery IQ	2 (2.63%)
Asteion	1 (1.32%)
Brilliance 64	1 (1.32%)
Brilliance Big Bore	1 (1.32%)
SOMATOM go.Now	1 (1.32%)
LightSpeed RT16	1 (1.32%)
Emotion 16 (2007)	1 (1.32%)
Sensation Cardiac 64	1 (1.32%)
Discovery STE	1 (1.32%)

Supplementary S3. Radiomic feature extraction configuration. Detailed parameters for image pre-processing and radiomic feature extraction.


```
imageType:
  Original:
    binWidth: 5
  Square:
    binWidth: 1
  SquareRoot:
    binWidth: 14
  Logarithm:
    binWidth: 26
  Exponential:
    binWidth: 1
  Gradient:
    binWidth: 2
  LoG:
    binWidth: 2
    sigma: [1.0, 2.0, 3.0, 4.0, 5.0]   Wavelet: {}
  LBP3D:
    binWidth: 1.0


featureClass:
  shape:
  firstorder:
  glcm:
    - 'Autocorrelation'
    - 'JointAverage'
    - 'ClusterProminence'
```

```
    - 'ClusterShade'
    - 'ClusterTendency'
    - 'Contrast'
    - 'Correlation'
    - 'DifferenceAverage'
    - 'DifferenceEntropy'
    - 'DifferenceVariance'
    - 'JointEnergy'
    - 'JointEntropy'
    - 'Imc1'
    - 'Imc2'
    - 'Idm'
    - 'Idmn'
    - 'Id'
    - 'Idn'
    - 'InverseVariance'
    - 'MaximumProbability'
    - 'SumEntropy'
    - 'SumSquares'
  glrlm:
  glszm:
  gldm:
  ngtdm:

setting:
  interpolator: 'sitkBSpline'
  resampledPixelSpacing: [1, 1, 1]
  padDistance: 10
```

minimumROIDimensions: 2

minimumROISize: 1

correctMask: true

voxelArrayShift: 1000

label: 1

Supplementary S4. Expanded patient characteristics. Comparison of baseline laboratory-based values between training and external validation cohorts.

	Total cohort	Training set	External validation	p
**Serum creatinase (median | IQR)**	0.8 (0.7-0.9)	0.8 (0.7-0.9)	0.9 (0.8-1.0)	0.195
**Alanine aminotransferase (median | IQR)**	27.0 (19.0-36.0)	29.0 (19.2-36.0)	23.0 (15.0-27.0)	0.170
**Aspartate transaminase (median | IQR)**	31.0 (25.0-43.0)	31.0 (25.0-46.8)	29.0 (25.0-34.0)	0.556
**Lactate dehydrogenase (median | IQR)**	229.5 (177.5-353.2)	242.0 (201.5-384.0)	163.0 (101.0-208.0)	<0.001
**Alkaline phosphatase (median | IQR)**	130.0 (96.2-188.0)	142.0 (101.0-200.0)	77.0 (69.0-130.0)	0.007
**Neutrophil count (median | IQR)**	4.6 (3.6-6.0)	4.7 (3.8-6.0)	4.3 (3.0-7.0)	0.591
**Platelet count (median | IQR)**	207.0 (158.5-256.8)	207.0 (158.0-254.0)	217.0 (160.0-260.0)	0.907
**Bilirubin (median | IQR)**	8.1 (1.0-12.5)	9.3 (4.5-12.9)	1.0 (0.7-1.1)	0.002
**Carcinoembryonic antigen (median | IQR)**	64.3 (19.2-265.8)	71.8 (20.0-279.2)	46.6 (19.0-197.0)	0.842
**Mismatch repair status (n | %)**				
**pMMR**	14 (18.4%)	14 (22.2%)	0 (0.0%)	0.124
**dMMR**	2 (2.6%)	2 (3.2%)	0 (0.0%)	
**Missing**	60 (78.9%)	47 (74.6%)	13 (100.0%)	

Supplementary S5. Final feature sets for imaging and clinical-lab models. The general radiomic signature, intensity features, and clinical-laboratory variables were included in the final machine learning models.

General radiomic signature	Intensity features	Clinical-lab features
original_firstorder_Maximum	original_firstorder_10Percentile	Gender
original_glcm_ClusterProminence	original_firstorder_90Percentile	Age
square_glszm_GrayLevelVariance	original_firstorder_Energy	ECOG performance
square_ngtdm_Strength	original_firstorder_Entropy	Dominant lobe affected
squareroot_firstorder_Kurtosis	original_firstorder_InterquartileRange	Synchronous vs. Metachronous
squareroot_glcm_ClusterProminence	original_firstorder_Kurtosis	Serum creatinase
logarithm_firstorder_Kurtosis	original_firstorder_Maximum	ALT
logarithm_firstorder_Range	original_firstorder_MeanAbsoluteDeviation	AST
logarithm_firstorder_Skewness	original_firstorder_Mean	LDH
logarithm_glcm_Autocorrelation	original_firstorder_Median	Alkaline phosphatase
logarithm_glrlm_LowGrayLevelRunEmphasis	original_firstorder_Minimum	Neutrophils
exponential_glrlm_ShortRunLowGrayLevelEmphasis	original_firstorder_Range	Platelets
exponential_glszm_SmallAreaHighGrayLevelEmphasis	original_firstorder_RobustMeanAbsoluteDeviation	CEA
exponential_gldm_DependenceVariance	original_firstorder_RootMeanSquared	RAS mutation
log-sigma-1-0-mm-3D_gldm_LargeDependenceLowGrayLevelEmphasis	original_firstorder_Skewness	BRAF mutation
log-sigma-2-0-mm-3D_firstorder_Mean	original_firstorder_TotalEnergy	Bilirubin
log-sigma-2-0-mm-3D_firstorder_Skewness	original_firstorder_Uniformity	MMR status
log-sigma-2-0-mm-3D_glcm_Correlation	original_firstorder_Variance	
log-sigma-2-0-mm-3D_glcm_Imc1		
log-sigma-3-0-mm-3D_firstorder_10Percentile		
log-sigma-3-0-mm-3D_firstorder_Median		
log-sigma-3-0-mm-3D_firstorder_Skewness		

log-sigma-3-0-mm-3D_glcm_ClusterShade		
log-sigma-3-0-mm-3D_glcm_Imc2		
log-sigma-4-0-mm-3D_firstorder_Median		
log-sigma-4-0-mm-3D_glcm_ClusterShade		
wavelet-LLH_glcm_ClusterShade		
wavelet-LLH_glszm_SmallAreaLowGrayLevelEmphasis		
wavelet-LHL_glrlm_LongRunLowGrayLevelEmphasis		
wavelet-LHL_glrlm_LowGrayLevelRunEmphasis		
wavelet-LHH_glcm_ClusterShade		
wavelet-LHH_glszm_SmallAreaLowGrayLevelEmphasis		
wavelet-HLL_glrlm_LongRunLowGrayLevelEmphasis		
wavelet-HLH_firstorder_Mean		
wavelet-HHH_firstorder_Mean		
wavelet-HHH_firstorder_Median		
wavelet-LLL_glrlm_ShortRunHighGrayLevelEmphasis		
lbp-3D-m1_firstorder_Maximum		
lbp-3D-m1_firstorder_Minimum		
